# Flow theory – goal orientation theory: positive experience is related to athlete’s goal orientation

**DOI:** 10.3389/fpsyg.2015.01499

**Published:** 2015-10-09

**Authors:** Nektarios A. M. Stavrou, Maria Psychountaki, Emmanouil Georgiadis, Konstantinos Karteroliotis, Yannis Zervas

**Affiliations:** ^1^Faculty of Physical Education and Sport Science, National and Kapodistrian University of AthensAthens, Greece; ^2^Sport Psychology Department, Hellenic Sports Research Institute, Olympic Athletic Center of Athens Spyros Louis College of Sports SciencesMaroussi, Greece; ^3^Department of Science and Technology, Faculty of Health and Science, University Campus SuffolkSuffolk, UK

**Keywords:** flow theory, goal orientation, challenge, skills

## Abstract

The main purpose of this study was to examine the relationship between flow experience and goal orientation theory, as well as, the differences in flow experience based on the orthogonal model of goal orientation theory. Two hundred and seventy eight athletes completed the Task and Ego Orientation Sport Questionnaire based on how they usually feel. The challenge and skills ratings were completed 1 h before the competition, based on how they felt at the exact time of answering. In the following, the Flow State Scale-2 was completed up to 30 min after the competition they just participated, along with the challenge-skill ratings, based on how athletes felt during the competition. The results indicated that the athletes’ task orientation may be an important factor for attaining flow in competitive sport, feeling more skillful and estimating the upcoming competition as challenging, while low ego and low task oriented athletes lack these elements, which are important for them to get into flow. Additionally, not the level of task and ego orientation *per se*, but the balance between athletes’ goal orientation preferences seems important for the formation of flow experience, indicating that high task – high ego and high task – low ego athletes are experiencing the most positive mental state.

## Introduction

As managing negative emotions has been the primary focus of practitioners in the past, positive emotions have received limited examination in the sporting context. Around the beginning of millennium this trend started to shift as a theoretical approach has emerged, under the name positive psychology (Seligman and Csikszentmihalyi, 2000). This new scientific field focused on the study of positive experience, trying to find the pathways to improve humans functioning, performance, and well-being. However, this approach is not new as three decades earlier Csikszentmihalyi (1982) started building this trend through the flow theory, examining ways to create and control this positive experience. The theoretical and practical links of positive experience to other well-established theoretical approaches, such as goal orientation theory will provide fruitful information to better capture the nature of optimal experience, which is helpful to consultants and coaches’ work, and athletes’ performance improvement.

### Flow Theory

Flow experience refers to an optimal and pleasurable experience, linked to intrinsically rewarding and high enjoyable feelings (Csikszentmihalyi and Csikszentmihalyi, 1988; Jackson and Csikszentmihalyi, 1999). In the sporting context, the athletes will experience flow when goals are clearly set by the athlete, feedback is immediate and unambiguous (Jackson and Csikszentmihalyi, 1999). This enables the athlete to be completely concentrated and absorbed by the activity, and to perceive the task at hand as self-rewarding, controllable and joyful. By adding the subsequent absence of worry about the upcoming result and the automatic and effortless experience, there is a great possibility that the athlete will experience flow (Csikszentmihalyi, 1988; Jackson, 1995; Jackson and Csikszentmihalyi, 1999).

The orthogonal model of flow theory constitutes an important part for understanding the quality of this enjoyable and self-rewarding subjective experience (Csikszentmihalyi and Csikszentmihalyi, 1988). Two characteristics formulate the quality of this experience: (a) self-perceived challenge of the situation or activity and (b) self-perceived skills of the person (Csikszentmihalyi, 1982; Moneta and Csikszentmihalyi, 1996). Moneta and Csikszentmihalyi (1996, p. 277) referred that the level of challenge represents the “intrinsic demands” of the activity, while the level of person’s skills refers to the “self-perceived capacity to meet the demands” of the activity. A key notion of flow experience is the perceived balance between challenge and skills, and not the objective nature of the challenges or skills *per se* (Csikszentmihalyi, 1982). According to Csikszentmihalyi (1988), a crucial prerequisite for an athlete to experience flow state, is the self-perceived or individually estimated balance of situational challenges and personal skills. When both challenge and skills are perceived as being in balance and above a person’s average, the athlete will experience an optimal state, which represents *flow*. However, significant individual and situational differences exist, indicating that in some cases individuals may experience flow in situations of low challenge (e.g., microflow; Csikszentmihalyi, 1975). On the other hand, when athlete’s skills exceed or do not fulfill the situational challenge, the athlete will experience different state(s). When an athlete perceives that own skills exceed perceived challenge of the activity, meaning a manageable challenge pertaining to the situation, the athlete will experience *relaxation*. On the other hand, when the challenge of the competition outweighs athlete’s skills, ensuing capacity subsides the demands of the task resulting in lower experience, leading the athlete to anxiety states. Finally, when both challenge and skills are perceived as low, and below a person’s average, the athlete will experience *apathy*, representing the worst subjective experience in the sporting context (Csikszentmihalyi, 1982; Jackson, 1995; Moneta and Csikszentmihalyi, 1996). The aforementioned states represent the four quadrants of the “orthogonal” model of flow theory (Csikszentmihalyi, 1982).

Associations between challenges and skills relative to flow experience and other psychological constructs incorporate theoretical and methodological issues that should be taken into consideration. In flow theory, one key methodological issue in testing challenges and skills is the operationalization of these variables as a single construct (e.g., FSS-2), or as two separate constructs (e.g., Experience Sampling Method, ESM; Csikszentmihalyi and Larson, 1987). The personal reflection on challenge should also involve an assessment of personal skills Engeser and Rheinberg (2008) noted. Engeser and Rheinberg (2008), and Koehn et al. (2013a) supported a more differentiated view on the interaction between athletes’ perception of challenge and skill and for testing predictions of the quadrant model. Fong et al. (2014), in recent meta-analysis, provided evidence that single-item measures of challenge-skills balance showed stronger correlations with global flow measures. In conclusion, the latest evidence on the importance of challenges and skills and the theoretical contentions of flow indicate that these constructs should be operationalised and evaluated separately, providing a more detailed information of the interplay among them.

With the orthogonal model of flow theory representing a central and important aspect of flow experience, it is useful to mention the appearing limitations of this approach. The disadvantages of the orthogonal model are that it is an approximate and vague classification system, referring to the flow quadrant (Moneta, 2012). Moneta (2012) mentioned that the orthogonal model of flow represents a simple classification system, which allows performing simple tests regarding the core nature of flow experience. However, the challenge – skill balance and ratings represent the most important and crucial elements in the formulation of flow experience (Csikszentmihalyi, 1982). Finally, all theoretical approaches such as the initial model of flow theory (Csikszentmihalyi, 1982), the quadrant model (Csikszentmihalyi and LeFevre, 1989), the experience fluctuation model (Massimini and Carli, 1988), the regression modeling approach (Moneta, 2012), and methodological approaches as the experience sampling method (Csikszentmihalyi and Larson, 1987) were characterized by several strength and weaknesses regarding theoretical and measurement limitations.

These limitations stress the quest for more definite predictions of flow states on the sporting environment. In a context were demands frequently fluctuate (Araujo et al., 2006) enriching flow examination with important motivational constructs can add significantly the ability to foster adaptive behavioral and emotional responses. Such a theoretical model is goal orientations theory (Nicholls, 1984, 1989).

### Goal Orientation Theory

Achievement goal theory (Nicholls, 1984, 1989) proposes that people approach achievement tasks with qualitatively different types of goals depending on how they are evaluating their competence and ability. Goal orientation theory, which is theoretically grounded on achievement goal theory, assumes that persons vary in the way they define accomplishment and judge perceived competence. Individual goal perspective influences how one thinks, feels, and acts in achievement situations such as education and sports.

Based on Nicholls’s theoretical work, Duda (1992) proposed the formation of two predominant achievement goal perspectives, namely *task orientation* and *ego orientation*, relating and differentiating how athletes estimate their level of ability, effort, and judgment of performance. In task orientation, ability evaluation is self-referenced, and is judged in relation to one’s own perceived mastery, understanding, or knowledge. Improving mastery and the execution of the task at hand remains the athlete’s major concern. In addition, perceived success is founded on personal improvement, skill learning, and effort exhibition. Overall, task orientation corresponds to adaptive cognitions and positive achievement behaviors, such as sustained involvement in athletic settings, practice, and optimal motivation, regardless of the person’s level of perceived ability (Duda, 1992, 2001).

On the other hand, an athlete is “ego oriented” when perceived ability is evaluated with regard to the performance of others implying a heavy reliance on social comparison for estimating personal ability. In this interpersonal comparison, outperforming the others with the least effort signifies superior competence (Nicholls, 1984, 1989; Duda, 1992). It is in this type of challenge that negative achievement behaviors (such as debilitated performance, deceptive tactics, reduced effort, and a lack of persistence) and maladaptive cognitive responses are assumed to result when individuals are ego-oriented and doubt their competence (White and Duda, 1994). It is supposed that an ego orientated athlete will show increased motivation only in conditions of high competence exceeding the demands of competition.

According to Nicholls (1989), Pensgaard and Roberts (2003), Roberts (2012) task and ego orientation are consistently thought to be orthogonal, and not bipolar. Research in education and sport settings supported the independence of goal orientation types. Examining the orthogonality of goal orientations Fox et al. (1994) examined four goal profiles [high task-high ego, high task-low ego, high ego-low task (HE-LT), and low ego-low task (LE-LT)] providing a different approach in the examination of the two goal perspectives. Results indicated that most highly motivated athletes were high task/high ego oriented, and the least motivated athletes were low task/low ego orientated, whereas the rest athletes were in between (Fox et al., 1994; Georgiadis et al., 2001).

### Flow Theory and Goal Orientation Theory: Is There a Theoretical and Research Link?

Research in flow theory has advanced considerably establishing a better understanding on how flow experiences appear. Researchers have been focusing on the personal elements, state characteristics, and psychosocial mechanisms involved in flow experiences (Kimiecik and Stein, 1992; Jackson, 1995; Jackson et al., 1998). Even though the studies examining the relevance of goal orientations and flow have been limited, theoretical propositions indicate a potential link between the two constructs (Csikszentmihalyi, 1988; Jackson and Roberts, 1992; Jackson and Csikszentmihalyi, 1999).

Having flow theory in mind, Kimiecik and Stein (1992) reported that being task-involved should enable athletes to focus more on the task at hand, indexing the ability to get absorbed in an activity. Consequently this could lead to the experience of flow characteristics (i.e., enjoyment of the activity), higher levels of concentration, and feeling in control. For example, athletes who continually compare their ability to others and are consumed by objective outcomes (win/loss) are more likely to perceive competitive situations as overly challenging which could lead to higher levels of anxiety. The flow literature and research would suggest that such a state is quite unlikely to produce flow experience or high levels of performance. This aspect is supported by Duda et al. (1990) goal orientations results, based on which increased levels of precompetitive state cognitive and somatic anxiety were primarily evident among high ego orientated athletes who did not expect to win the competition at hand. Jackson (1988), as well as Jackson and Roberts (1992) proposed that athletes with a task orientation may experience flow more frequently compared to athletes with an ego orientation, or with a low task involvement, since they perceived their ability as higher, which serves as an important factor for positive experience. Further to the above, Jackson (1988) mentioned that ego-oriented athletes, particularly in the case of low perceived ability tend to experience flow less frequently when competing.

Providing further support to the theoretical link between flow and goal orientation theories, van de Pol and Kavussanu (2011) indicated that task orientation is positively related to enjoyment, which is closely linked to flow, whereas ego orientation provides negative correlation to enjoyment and overall intrinsic motivation (Duda et al., 1990). Similarly, Duda et al. (1995) indicate that task-involved athletes experience greater intrinsic interest in tasks, and that are more likely to be performing the task for its own sake which both refer to characteristics of flow experience. Also in a physical education context, Papaioannou and Kouli (1999) argue that the existence of task orientation and task orientation climate predicted higher levels of concentration, autotelic experience, and loss of self-consciousness. In the same vein, Kowal and Fortier (1999) referred that situational self-determined forms of motivation (e.g., task-involvement) may facilitate flow, whereas non-self-determined forms of motivation (e.g., ego-involvement) may have a detrimental influence on flow states. Finally, Biddle et al. (2003), reviewed 47 studies, regarding the association of goal orientation and positive affect, which was mainly operationally defined as enjoyment, and satisfaction, noting that the vast majority (89%) of the research findings supported a positive relationship of positive affect and task orientation.

In sport psychology, it is a commonplace that research has focused mainly on the negative factors of athlete’s experience (e.g., anxiety), ignoring the positive psychological qualities that improved performance is liaised with. However, the competitive environment continuously presents athletes with opportunities for action or new challenges to manage providing an environment in which personal skills are required to master these challenges. A sense of competence results from balancing challenge and skill, and the type of goal orientation is believed to underlie this process. With this in mind, the theoretical relation of flow experience and goal orientations has been sparsely examined. Tackling such a theoretical need will add clues on the independent relation of flow and goal orientation, and mainly on the consonance of ego-task and challenge-skill ratios dimensions. As these orthogonal models which denote different theoretical views create a key field producing an interesting theoretical synthesis (Biddle et al., 2003), that could provide a more comprehensive explanation of the sporting environment and its underlying adaptive athletic experience. Such a synthesis to the best of our knowledge has not been attempted yet, constituting a restriction in the examination of athletes’ experience in the sporting context.

More precisely the reasons justifying the importance and the usefulness of the concurrent examination of flow experience and goal orientations are related to the fact that previous theoretical suggestions connecting the variables under examination were based on cross-sectional examinations of responses lacking the important elements of temporal change and real time assessment (i.e., Jackson et al., 1998; Koehn et al., 2013b), both of them deemed important based on the theoretical analysis of flow (Csikszentmihalyi and LeFevre, 1989). Such studies have measured the relationship between goal orientation and flow experience based on a retrospective measure whereas a large number of flow studies applied a retrospective design measuring flow state once after the event or based on athletes’ past experience (i.e., Jackson and Roberts, 1992). Added to that, although previous research has examined the link of goal orientation and positive experience (i.e., enjoyment, pleasure, peak experience; Duda et al., 1990; van de Pol and Kavussanu, 2011), however, these elements are not identical to flow experience as they hold distinct characteristics. Hence, should not be at the forefront of such an analysis (Privette and Bundrick, 1991; Jackson, 2000). Finally, several methodological concerns have been raised in previous research in the flow experience measurement. Previous studies used single item scales characterized by methodological limitations or partially measured flow experience in an effort to capture flow characteristics (Jackson and Roberts, 1992). The current study uses a well-documented instrument designed to fully capture the flow concept tackling this methodological issue.

Previous research findings suggested a theoretical link between goal orientation and positive emotions through mere associations among singled variables. Attempting to examine the not yet explored link between the orthogonal model of flow experience and its relation to the orthogonal nature of task and ego orientations could provide useful information on the interplay and the interaction of these theoretical models. Since the two theories indicate that it is not the relationship of each scale *per se*, but the balance and the interaction between the two theoretical approaches, this exploration could add fruitful information creating a better understanding on how athletes feel before and during competition. In the same venue, previous theoretical proposals among these constructs were based on correlational analyses (Duda et al., 1990; van de Pol and Kavussanu, 2011). Employing more rigorous statistical methods can provide clear and graphical associations among variables in focus (i.e., correspondence analysis), and pinpoint any differences among athletes of distinct motivational vignettes (high task-high ego, high task-low ego, HE-LT, and LE-LT). Hence, the purpose of the current research is to examine (a) the nature of the relationship between flow experience and goal orientations in a more accurate and elucidatory way, (b) the differences in flow experience based on the orthogonal model of goal orientation theory [LE-LT, HE-LT, low ego-high task (LE-HT), high ego-high task (HE-HT)], and (c) the relationship between the orthogonal models of flow and goal orientation theories. Given the theoretical postulation underlying flow theory and goal orientation theories, it was hypothesized that task orientation will be positively related to flow experience and challenge – skills ratings, whereas non-significant correlations will appear between ego orientation with flow and challenge – skills scales (H1). Additionally it was hypothesized that significant differences will be revealed in FSS-2 factors and challenge – skills ratings among the goal orientation quadrants, with high task groups (LE-HT, HE-HT groups) indicating a higher flow experience (H2). Finally, it is hypothesized that significant relationships will appear between flow and goal orientation orthogonal model quadrants (H3).

## Materials and Methods

### Participants

Two hundred and seventy two athletes (n_male_ = 160 and n_female_ = 112) volunteered to participate in the study. Respondents were drawn from sports clubs and national teams participating in seven individual sports. Athletes participated in track and field (n = 89); swimming (n = 53); shooting (n = 14); archery (n = 14); tae-kwon-do (n = 8); skiing (n = 5); cycling (n = 77); table-tennis (n = 1); canoe-kayak (n = 11); and fencing (n = 6). Athletes’ age ranged from 16 to 35 years (M_age_ = 19.47 years, SD = 3.74), with their competitive experience ranging from 2 to 15 years (M_experience_ = 6.37 years, SD = 3.78). Finally, athletes’ participation in official competitions of national and international levels ranged from 10 to 350, with a mean of approximately 80 (SD = 67.97) competitions (Md = 60).

### Instrumentation

#### Flow Experience

The Flow State Scale-2 (FSS-2; Jackson and Eklund, 2002; Stavrou and Zervas, 2004) was used in the present study. The FSS-2 comprised of 36 items developed to measure the quality of flow experience during athlete’s participation in the competition, indicating acceptable construct validity and reliability [χ^2^/df ratio = 2.56, Non-Normed Fit Index (NNFI) = 0.901, Comparative Fit Index (CFI) = 0.912, Root Mean Squared Error of Approximation (RMSEA) = 0.052, 90% CI of RMSEA = 0.049–0.055)]. Also, the Greek version of the FSS-2 indicated acceptable factor as this was examined through confirmatory factor analysis [χ^2^/df ratio = 2.97, NNFI = 0.903, CFI = 0.914, Robust CFI = 0.925, Standardized Root Mean Squared Residual (SRMR) = 0.061, RMSEA = 0.054, 90% CI of RMSEA = 0.051–0.057] and reliability (Cronbach α = 0.75–0.92). The 36 items constitute the following nine qualities that typify the factors of the FSS-2: (a) challenge-skill balance, (b) action-awareness merging, (c) clear goals, (d) unambiguous feedback, (e) concentration on task at hand, (f) sense of control, (g) loss of self-consciousness, (h) transformation of time, and (i) autotelic experience.

#### Goal Orientation

The Task and Ego Orientation in Sport Questionnaire (TEOSQ; Duda, 1989) is a 13-items sport-specific instrument that measures ego orientation and task orientation. Through confirmatory factor analytic procedures, the TEOSQ has showed acceptable validity and reliability indices in the original version in various samples (Goodness Fit Index ≥ 0.89, CFI ≥ 0.83, SRMR ≤ 0.09; Chi and Duda, 1995). The TEOSQ has been adapted to Greek population (Karteroliotis and Stavrou, 1996) providing acceptable factor structure (χ^2^/df ratio = 2.92, NNFI = 0.902, CFI = 0.910, RCFI = 0.917, SRMR = 0.097, RMSEA = 0.088, 90% CI of RMSEA = 0.081–0.097) and internal consistency (Cronbach α = 0.80–0.83).

#### Challenges and Skills Measures

Two 11-point scales were administered to measure the challenge of the competition, and the perceived level of athlete’s skills. Assessing levels of challenges and skills as separate constructs has been conceptually supported by Csikszentmihalyi (1975) and Jackson and Marsh (1996), and applied by Stavrou et al. (2007). On hour before the scales’ wording was the following: (a) “How challenging is this event for you?”, measuring the perceived challenge of the competition, and (b) “How skilled do you feel for this event?”, measuring the perceived skills of the athlete. For the “just after the competition” time measure the scales’ wording changed to the following: (a) “How challenging was this event for you?”, and (b) “How skilled did you feel in this event?”. The anchors for the two scales ranged from 0 (*not at all*) to 10 (*very much*), with a midpoint of 5 (*medium*). Similar one-item scales examining perceived competition challenge and athletes’ skills have been used by Csikszentmihalyi and Larson (1987), Stein et al. (1995), Jackson et al. (1998), Stavrou et al. (2007), Abuhamdeh and Csikszentmihalyi (2012).

### Data Analysis

Multivariate relationship between goal orientation and flow experience was examined through canonical correlation analysis. Canonical correlation aims to analyze the association between two composite sets of variables (Tabachnick and Fidell, 2006). The predictor variable set comprised of ego and task orientation, and the criterion variables set contained the nine FSS-2 subscales. Pearson’s *r* correlation coefficient was used to examine for relationships among the examined variables.

Additionally, univariate and multivariate statistical analyses were conducted in order to examine whether athletes in the four goal orientation quadrants differed significantly in the FSS-2 subscales during competition. Follow-up ANOVAs were performed on the subscales where there were significant MANOVA effects (Tukey test). Bonferroni adjustment was applied to control for the inflation of Type I error (Tabachnick and Fidell, 2006).

Although several researchers have criticized and not encouraged the dichotomization of quantitative variables (MacCallum et al., 2002) due to loss of power or increase of Type I error, however, dichotomization might be used in some cases without methodological concerns (DeCoster et al., 2009). Based on the purpose of the study, we decided to dichotomize the challenge-skill dimension of the flow model and the task and ego orientation based on the theoretical suggesting of the flow and goal orientation theory. Another issue of the study was to examine how the dichotomized variables will perform in the field, providing practical information regarding the match of the two theoretical approaches (DeCoster et al., 2009). Finally, it seems to be easier to analyze and interpret categorical data, having in mind the theoretical approach of the present study, since correspondence analysis was used to graphically represent the relationship among the quadrants of the two orthogonal models of flow and goal orientation theories.

Correspondence analysis is a non-parametric statistical technique used to depict the relationships among two categorical variables (Benzécri, 1992; Sourial et al., 2010; Doey and Kurta, 2011; Garson, 2012; Glyn, 2013). Correspondence analysis provides also a visual result of the relationship between the examined categorical variables, through a multidimensional graphical map (Hoffman and Franke, 1986; Garson, 2012). In this map, two- or multi-way tables with each row and column are becoming a point on a multidimensional graphical map, called a biplot. These points are produced as a result of the row and column analysis in the contingency table data, as a result of nominal values, of no particular order (Glyn, 2013). The distance between row and column points provides a graphically view of similarities or differences among the variables/categories. Points that are placed close to one another have similar profiles, whereas pointed mapped away represent different profiles, providing a holistic overview of the data trends that facilitates the detection of relationships (Sourial et al., 2010).

With regard to the above, correspondence analysis was chosen as the most appropriate technique for examining the associations between the two multi-level categorical variables of the current study providing information on how the variables are related, and not if a relationship exists among them or not (Hoffman and Franke, 1986). Symmetrical normalization was used to standardize row and column data points, for examining the relationship among the variables; thus, the general comparisons between them can be made (Doey and Kurta, 2011). None of the assumptions pertaining to correspondence analysis was violated (Doey and Kurta, 2011; Garson, 2012). Specifically, no empty and non-negative entries were appeared in the frequency contingency table and, the variables that were examined were discrete, with no specific order. Additionally, there were four categories for the two examined variables, producing a high complex contingency table, serving as reason for choosing correspondence analysis as the preferable method of analysis, instead of others (e.g., log-linear analysis; Garson, 2012).

### Procedure

Following approval by the University Ethics Committee, we requested access to athletes with a sport background who were actively competing in their sport (participating in trainings and competitions). The athletes were informed about the purpose of the study, the assessment, and the data collection procedure. Oral information was provided by the main researcher, which was present during the instrument completion, explaining what the instruments were about, how they will complete them, and the procedure that will be followed. The written information contained an introductory section in each instrument that the athletes had to read it before moving the items responses. They were reassured that their responses would be kept strictly confidential and that they are going to be used only for research purposes. The athletes were asked to voluntarily participate and they completed a consent form prior to questionnaire completion, and were tested individually or in small groups. Researchers recruited the athletes either through personal contact or through their coaches, visiting them at their training venues.

TEOSQ was completed in a non-competitive situation (i.e., training, sport psychology lab). The challenge and skills ratings were completed 1 h before the competition, based on how they felt at the exact time of answering. Additionally, the FSS-2 was completed up to 30 min after the competition they just performed, along with the challenge-skill ratings, based on how athletes felt during the particular competition. The retrospective measure of flow has been criticized for conceptual reasons (Rheinberg, 2008). However, this type of measure is preferred instead of others (e.g., experience sampling method) when the main research interest is focused on the relationship of flow with other psychological constructs (Schüler and Brunner, 2009). The instructions for the instruments were modified in an adequate format for use before or after the competition. Data collection lasted approximately 1 year.

## Results

### Preliminary Analysis

#### Data Screening

Univariate and multivariate distribution analyses were performed prior to data analyses (Tabachnick and Fidell, 2006). Skewness and kurtosis indicated low values of the examined variables of TEOSQ and FSS-2 (skewness_range_ = -1.01 to 0.06, kurtosis_range_ = -0.75 to 0.77). Examination of Mahalanobis distance values indicated six multivariate outliers (*p* < 0.001) among the independent variables, that were excluded from further analysis. At the univariate level, the equality of covariances matrices was non-significant (Levene’s test, *F*_max_ ratio values), and at the multivariate level the homogeneity of variance–covariance was acceptable (Box’s *M*-test).

### Main Analysis

#### Flow Experience and Goal Orientation Relationship (H1)

In the canonical correlational analysis the ego and task orientation were the prediction variables with the criterion variables containing the nine FSS-2 subscales. The canonical correlation analysis yielded two functions with canonical correlation (*R*_c_; **Table 1**). The canonical correlation of *R*_c1_ was 0.36 and *R*_c2_ was 0.25. The overall multivariate model across the two functions (Functions 1 to 2) was statistically significant, Wilk’s Λ = 0.82, *F*(18,522) = 3.14, *p* < 0.001. The dimension reduction analysis was used to test the statistical significance of the hierarchical arrangement of functions. Function 2 was also significant, Wilk’s Λ = 0.94, *F*(8,262) = 2.26, *p* < 0.05. Although Function 2 did not meet the cut-off criterion of 0.30 for *R*_c_ selection (Tabachnick and Fidell, 2006; p. 587), two functions were selected for practical significance and reasons of interpretation (Hair et al., 1998). The proportion of shared variance (*R*_c_^2^) between the criterion variable set and the prediction variable set for the full model (across the two functions) was 0.19%. According to Tabachnick and Fidell (2006) canonical loadings ≥0.30 are considered meaningful for interpretation. In the first canonical function, seven out of nine FSS-2 factors revealed a significant positive loading on the flow experience variate. The correlation (*R*_c1_ = 0.36) between the two canonical variable sets indicate that athletes who experience flow are mainly task oriented, and secondly ego oriented. In the second canonical function, the results indicated that ego-oriented athletes didn’t show high levels of flow experience, however, the relationship between the two variable sets was rather low (*R*_c2_ = 0.25). The results provided support to the Hypothesis 1.

**Table 1:** Canonical correlation analysis of goal orientations and flow experience.

	Function 1	Function 2
Variables	Canonical loadings	Canonical loadings
**Criterion set**		
Challenge-skill balance	0.62	0.59
Action-awareness merging	0.13	0.55
Clear goals	0.80	0.20
Unambiguous feedback	0.69	0.31
Concentration on task at hand	0.59	0.25
Sense of control	0.53	0.45
Loss of self-consciousness	0.06	0.67
Transformation of time	0.34	-0.09
Autotelic experience	0.50	0.74
Percent of variance	0.28	0.23
Redundancy	0.10	0.06
**Predictor set**		
Ego orientation	0.67	-0.74
Task orientation	0.86	0.51
Percent of variance	0.59	0.41
Redundancy	0.21	0.10
Canonical correlation	0.36	0.25
Squared correlation	0.13	0.07
*p* <	0.001	0.05

Task orientation revealed medium to low positive correlations with competition challenge (1 h before: *r* = 0.24, *p* < 0.001, during competition: *r* = 0.25, *p* < 0.001) and athletes’ skills (1 h before: *r* = 0.24, *p* < 0.001, during competition: *r* = 0.25, *p* < 0.001). Ego orientation indicated low positive correlation with competition challenge (1 h before: *r* = 0.20, *p* < 0.001, during competition: *r* = 0.18, *p* < 0.01), and low to non-significant correlation with athlete’s skills (1 h before: *r* = 0.15, *p* < 0.05, during competition: *r* = 0.10, *ns*). The Hypothesis 1 was partially supported from the research findings.

#### Differences in Flow Experience based on Goal Orientation Model (H2)

Four groups were formulated based on the scales of task orientation and ego orientation median splits. Group 1 (*n* = 63) consisted of the low ego and low task oriented athletes. Athletes in Group 2 (*n* = 64) were high in ego, and low in task orientation, whereas the members of Group 3 (*n* = 55) comprised of low ego and high task oriented athletes. Finally, Group 4 (*n* = 90) consisted of athletes high in both ego and task orientation. The multiple analysis of variance results, Pillai’s Traice = 0.174, *F*(3,268) = 1.79, *p* < 0.01, $\eta_{p}^{2}$ = 0.06, indicated significant differences between the goal orientation groups in the FSS-2 subscales (**Table 2**). Follow-up univariate ANOVAs revealed significant differences in the following FSS-2 factors: challenge-skill balance *F*(3,268) = 6.31, *p* < 0.001, $\eta_{p}^{2}$ = 0.07, clear goals *F*(3,268) = 5.06, *p* < 0.01, $\eta_{p}^{2}$ = 0.05, unambiguous feedback *F*(3,268) = 5.27, *p* < 0.01, $\eta_{p}^{2}$ = 0.06, sense of control *F*(3,268) = 4.40, *p* < 0.01, $\eta_{p}^{2}$ = 0.05, and autotelic experience *F*(3,268) = 6.91, *p* < 0.001, $\eta_{p}^{2}$ = 0.07. Finally, analysis of variance revealed significant differences among the goal orientation groups in challenge of the game *F*(3,268) = 5.05, *p* < 0.01, $\eta_{p}^{2}$ = 0.05, and athletes’ skills *F*(3,268) = 4.03, *p* < 0.01, $\eta_{p}^{2}$ = 0.04. The findings showed that high task oriented athletes experienced higher flow experience, providing support to Hypothesis 2.

**Table 2:** Means (*M*), standard deviations (*SD*) in the Flow State Scale-2 subscales based in the high/low task and ego orientation athletes.

	LE-LT		HE-LT		LE-HT		HE-HT	
	1		2		3		4	
Flow State Scale-2 subscales	*M*	*SD*	*M*	*SD*	*M*	*SD*	*M*	*SD*
Challenge-skill balance^a-b-d^	3.18	0.81	3.34	0.81	3.78	0.74	3.58	0.88
Action-awareness merging	3.11	0.76	3.06	0.71	3.46	0.75	3.17	0.87
Clear goals^b-d^	3.63	0.83	3.81	0.66	4.01	0.72	4.07	0.78
Unambiguous feedback^b-d^	3.04	0.86	3.25	0.75	3.57	0.83	3.48	0.89
Concentration on task at hand	3.50	0.83	2.53	0.79	3.85	0.76	3.81	0.91
Sense of control^b-c^	3.42	0.78	3.46	0.70	3.88	0.70	3.71	0.93
Loss of self-consciousness	3.28	0.91	3.11	0.92	3.64	0.82	3.29	1.07
Transformation of time	2.77	0.84	3.09	0.84	3.05	0.90	2.95	0.86
Autotelic experience^b-c-d-e^	3.14	1.12	3.15	1.13	3.89	1.06	3.63	1.12
Challenge of the game^d-e^	6.63	2.26	7.02	2.43	7.36	2.53	8.08	2.08
Skills of the athlete^b-d^	6.35	2.36	6.95	2.07	7.58	2.03	7.46	2.41

*LE, Low ego orientation; LT, Low task orientation; HE, High ego orientation, HT, High task orientation.*

*^a^Group 1 significantly lower than Group 2, ^b^Group 1 significantly lower than Group 3, ^c^Group 2 significantly lower than Group 3, ^d^Group 1 significantly lower than Group 4, ^e^Group 2 significantly lower than Group 4.*

#### Correspondence Analysis (H3)

Using correspondence analysis, the relation between the orthogonal models of flow (apathy, anxiety, relaxation, flow) and goal orientation theories (LE-LT, HE-LT, LE-HT, HE-HT) groups, was examined. The row and column variables represent the four quadrants of the two orthogonal models of flow and goal orientation theory indicating a significant correspondence (Chi-square χ^2^ = 29.79, df 9, *p* < 0.001; **Table 3**).

**Table 3:** Flow theory quadrants by goal orientation theory quadrants contingency table.

	Apathy	Anxiety	Relaxation	Flow	Total
L/E–L/T	30	11	8	14	63
% Goal quadrant	47.6%	17.5%	12.7%	22.2%	100.0%
% Flow quadrant	37.0%	22.0%	18.2%	14.4%	23.2%
H/E–L/T	25	10	8	21	64
% Goal quadrant	39.1%	15.6%	12.5%	32.8%	100.0%
% Flow quadrant	30.9%	20.0%	18.2%	21.6%	23.5%
L/E–H/T	8	11	16	20	55
% Goal quadrant	14.5%	20.0%	29.1%	36.4%	100.0%
% Flow quadrant	9.9%	22.0%	36.4%	20.6%	20.2%
H/E–H/T	18	18	12	42	90
% Goal quadrant	20.0%	20.0%	13.3%	46.7%	100.0%
% Flow quadrant	22.2%	36.0%	27.3%	43.3%	33.1%
Total	81	50	44	97	272
% Goal quadrant	29.8%	18.4%	16.2%	35.7%	100.0%
% Flow quadrant	100.0%	100.0%	100.0%	100.0%	100.0%

*L/E–L/T, Low ego–low task athletes; H/E–L/T, High ego–low task athletes; L/E–H/T, Low ego–high task athletes, H/E–H/T, High Ego–high t ask athletes.*

The total inertia of the correspondence analysis was 0.110. The number of dimensions (principal axis) drawn from the analysis were three, which represent the number of variables categories on row and columns minus 1 (I–1, J–1) of the contingency table. The first dimension accounted for the 78.0% proportion of inertia (λ_1_ = 0.085), the second for 21.3% of the total variance (λ_2_ = 0.023), whereas the third dimension accounted for only 0.7% (λ_3_ = 0.001). Two dimensions were kept based on the criteria of the percentage of explained variance over 70%, and the percentage of explained variance by each dimension over 20% (Higgs, 1991; Hair et al., 1998). Correspondence analysis indicated that all points contributed to the dimension over 50%, which is the cut-off criterion for keeping or retaining a point of the joint plot, based on Hair et al. (1998) suggestions. Specifically, the row points (goal orientation) contribution to the dimension ranged from 58 to 96%, whereas for the column points (flow) contribution the range was from 52 to 99%.

**FIGURE 1:**
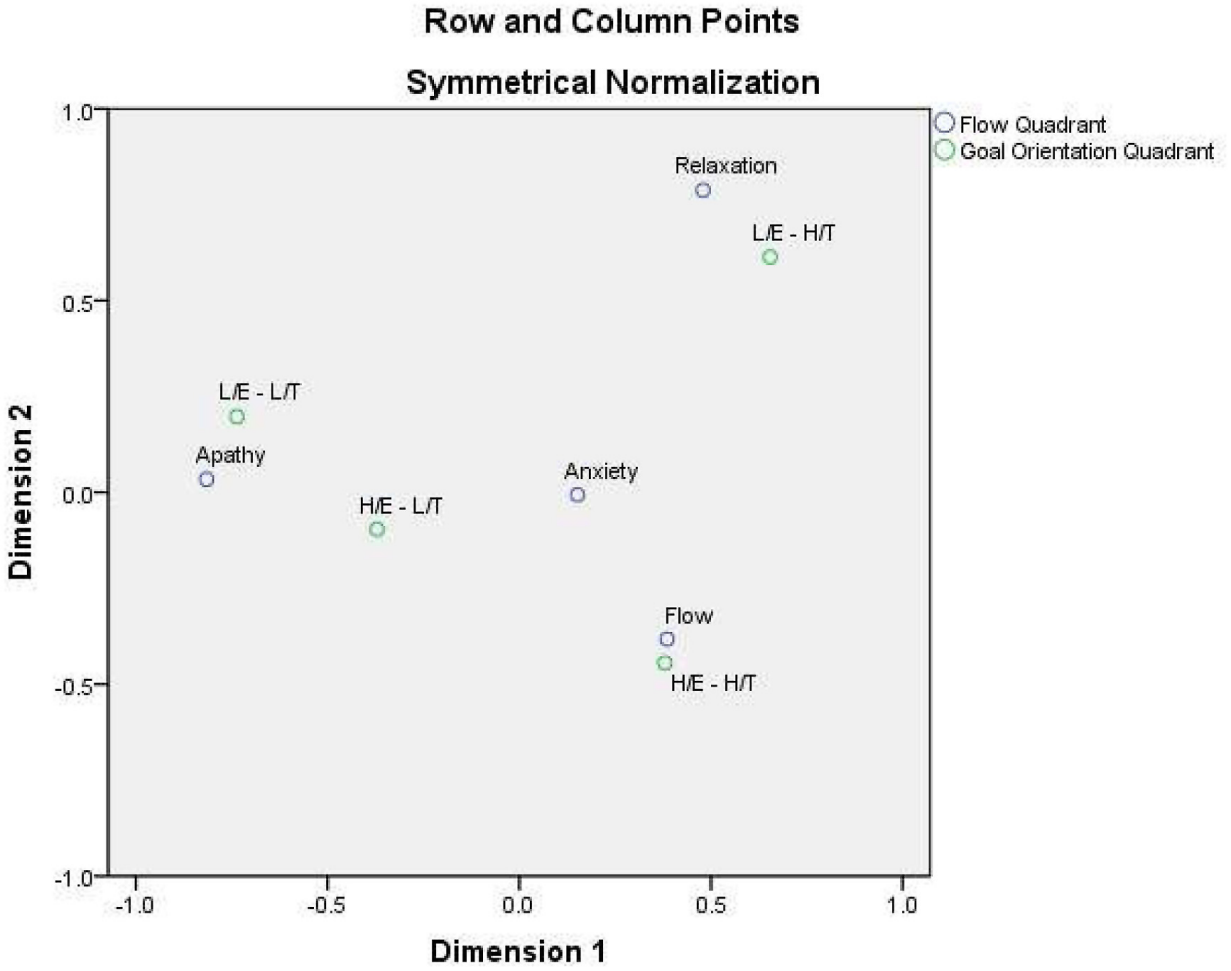
**Correspondence analysis map of flow theory and goal orientation theory groups**.

Dimension 1 is presented in the horizontal axis, whereas Dimension 2 relates to the vertical axis. To visualize the associations of column and row points on a two-dimensional space, a correspondence map analysis is displayed graphically in a biplot in **Figure 1**. In the biplot, the flow group clusters with HE-HT group and the relaxation group maps close to LE-HT group athletes, with each of the dyads falling into the same quadrant. Similarly it is clear that the LE-LT group athletes falls closer to the apathy group and both of them in the same quadrant. Finally the HE-LT group of athletes’ falls between the apathy, anxiety, and flow group, indicating that the specific goal orientation group might fall close to one of the three aforementioned goal groups, depending on the situational and personal characteristics. Based on the aforementioned findings, support was provided to the third Hypothesis. Although correspondence analysis does not define mathematically the distances between the four categories of the flow and goal orientation groups, the distance between any row points or column points gives a measure of their similarity (or dissimilarity). The proximity of two points in a correspondence map or the fact that two points are represented in the same quadrant indicates similar profiles and points of row and column variables closely related (Higgs, 1991; Garson, 2012).

## Discussion

### Goal Orientation and Flow Experience Relationship (H1)

The importance of task orientation on the quality of the examined sporting experience seems to be supported by the higher correlations among the FSS-2 factors and task orientation, compared to the correlations of the FSS-2 factors and ego orientation, denoting a more proximal relationship between task orientation and flow factors. In other words, during competition, a task oriented athlete will feel enabled to meet the challenge of the game, to have clear goals, to enjoy participation and to receive immediate and unambiguous feedback on the level of performance. Also, high task orientation seems to have a positive relation to other qualities of experience, such as concentration, and sense of control over the activity, while ego orientation seems to be irrelevant to flow experience. This finding is supported for the first time in literature. Consequently such conclusions provide support to the notion Jackson and Roberts (1992) argued about relevant to task orientation and its close relation to flow experience.

Another important finding of the current study is that based on the examined associations, challenge and skills did not have a direct and close relationship to goal orientations (a result held true for both ego and task orientations). This suggests that other psychological or environmental characteristics may mediate these relations, (i.e., perceived competence and personal goals) and could add explanatory power to the suggested relationship. Regarding the two time measures of athletes’ skills and situational challenge, the value correlations between the task orientation and those of game’s challenge and athlete’s skills remained stable, while the correlations between ego orientation and challenge of the situation and skills of the athlete were lower and decreased across the two consecutive measures. This might mean that the relation between ego orientation and flow is rather unstable depending on situational factors (e.g., difficulty of opponent, opportunity to win), whereas on the other hand the relation between task orientation and flow seems to remain stable across time. This enhances the value and shows the significance of the stable sense of competence (e.g., based on personal improvement). Taking into consideration the independent relationship, as well as the ratio between challenge – skills and ego – task orientation it may be the balance between them that formulate athletes’ flow experience and not just their independent relation.

### Flow Experience Differentiation Based on Athlete’s Goal Orientation (H2)

The quality of experience differentiates between the four quadrants of the orthogonal model of goal orientation. The athletes in the HE-HT and LE-HT quadrants experienced significantly higher flow characteristics, compared to the athletes in the HE-LT and LE-LT quadrants, with the latter having significantly lower positive experience. This finding suggests that it is the task orientation level that is deemed more important than ego orientation for getting into flow in a competitive environment. In other words, high task orientation irrespectively, of the ego orientation level (either low or high), relates to the athletes’ efficiency to meet the demands of the task and the experience of a sense of control over effort and performance (Cumming and Hall, 2004). This can help athletes to concentrate on the task at hand, eliminating irrelevant cues or errors, and providing the opportunity for a strong and immediate sense of performance quality; all of these are important for an athlete to be in a flow state. This result extends the research in flow theory, indicating that being task-involved corresponds to the athletes’ flow experience, whereas a detrimental or neutral effect is denoted by a strong ego involvement.

Having the advantage of basing the conclusions of this study on real-time measurements of results and associations, the level of the athlete’s task orientation may be the primary factor for one’s positive experience or optimal mental states, whereas ego orientation seems to have a facilitative role only when athlete’s task orientation is sufficiently increased (Duda, 2001; Wang and Biddle, 2001; Cumming et al., 2002). From a practical point of view, coaches, athletes, and sport psychology consultants should focus on athletes’ level of task orientation during sport activities (both training and competitions) and factors related to self-determined and self-referenced comparisons, such as personal goals, improvement of athlete’s performance. At the same time they need to avoid emphasizing winning or outperforming opponents. Aiming toward this end, psychological preparation programs could include individual goal setting program, self-evaluation strategies and self-referenced criteria of success. The product of these techniques could contribute to increased competence levels sustaining motivation and extending sport participation.

### Flow and Goal Orientations Models Relationship (H3)

The previously mentioned results can be further supported by the differences in challenge and skills ratings between the four goal orientation quadrants, as well as, the correspondence analysis map. The athletes who experienced flow during the competition were mainly these with a high ego and task orientation, whereas the athletes who were in relaxation, based on flow theory, cluster close to the athletes with a HT-HE orientation as this was also supported by the mapping display. Finally, the athletes grouped in the apathy quadrant were those that were both low in ego and task orientation. These results showed that the subjective ratio characterized the flow and goal orientation models might be critical for the relation between goal orientation and flow experience.

The connotation of the previous finding suggests that any differences among the goal orientation quadrants in challenge and skill rates, as well as the relationships between the dimensions of flow and goal orientations orthogonal models, are related to the interaction rather the independent relationship between the dimensions of the two theories. Hence, it is the task rather than the ego orientation level that is more important for an athlete to estimate a competitive situation as challenging, and rate oneself as skillful enough. Both of these psychological states are known as important elements for one to get into flow (Duda, 2001; Wang and Biddle, 2001; Cumming et al., 2002; Jones et al., 2009). It also seems that a high task oriented athlete feels more skillful, whereas a low task oriented one feels less abled irrespectively of the level of ego orientation. Such finding shows that no matter what the level of ability (within reason) of a task involved athlete’s opponent, one will attempt to match challenges with skills, sometimes creatively, in order to learn the most from the situation and perhaps get into flow.

### Limitations

Certain limitations of the current study should not be overlooked. First, we examined the theories from a quantitative perspective. Trying to quantify athlete’s flow experience has certain limitations, since it cannot portray the subjective nature of flow phenomenon (Csikszentmihalyi, 1982). Qualitative research would be useful for enlightening the formulation of flow experiences within a sport setting, and its relationship to an athlete’s goal orientations. Also, the theoretical limitations proposed regarding the orthogonal model of flow theory (Rheinberg, 2008; Moneta, 2012), as well as, the dichotomization of quantitative variables (MacCallum et al., 2002) should be taken into consideration. Finally, the findings of the current study should be treated with caution as solely athletes of individual sports participated in the study. Flow experience seems to be affected by environmental factors such as type of sport (Jackson, 1992), so the current findings might not have an application in team sports.

## Conclusion

An important practical implication of the current study is that the differences found in flow experience based on goal orientation quadrants, supported the notion that being focused on the task and having self-referenced criteria for success seem to be crucial for an athlete to feel skillful enough to manage the demands of the competition. This type of orientation is important for estimating the upcoming competition as challenging, and not threatening. This could enable competitors to master sporting challenges leading to flow state. On the other hand, feelings of anxiety or apathy are quite possible, because an ego-involved athlete may perceive opponents as threatening or as less challenging than they really are to protect perceptions of competence. That is, they may underestimate the challenge, and act as if the competition is rather a bore drill (i.e., unworthy). This challenge could also be seen as overly difficult contributing to reduced effort and negative experiences such as apathy or anxiety. Providing support to the above, the theory of challenge and threat states in athletes (TCTSA; Jones et al., 2009) mentioned the importance of goal orientation, along with self-efficacy and sense of control, in the formulation of challenge and threat states. According to this theory, mastery approach goals reflect a motivation driven by self-referenced criteria helping the athletes to appraise an upcoming competition as challenging, whereas performance approach goals are more closely linked to a threat appraisal of sport competition, providing support to the results we found.

Based on the overall findings of the current study, the level of perceived challenge of the game refers to a cognitive evaluation, which is based on (or modulated by) athletes’ expectations (or goals) as well as ones’ perceived ability to manage competition demands that is linked to goal orientations. On the other hand, how skillful an athlete feels about an upcoming performance is based on an evaluation of personal efficiency and skills, especially in relation to the level of difficulty of the competition or the opponent. This evaluation is also highly dependent on the athlete’s ego or task orientation (Cumming and Hall, 2004). An unanticipated event, such as an injury, an opponent competing contrary to expectations at high standards, a non-anticipated win, or a focus on social evaluation during the game, could be associated with changes on the perception of competition challenge, or self-evaluation of the athlete’s skills. A competition may become frightening for an ego oriented athlete hindering performance due to the unmanageable demands of the situation (i.e., a difficult opponent). The same competition can be estimated as challenging by a task-dominant athlete through the opportunity to improve own capabilities as the challenging situation can be seen as an opportunity to extend personal skills. In relation to the four experiential states of the orthogonal model of flow theory, there is a suggestion that an athlete’s experience can be highly situational dependent for an ego oriented athlete but not for a task oriented one. For this reason it seems important to measure situational goal orientations, instead of, or in combination to the dispositional goal orientations measurement.

In summary, the current study had the advantage of examining a real-time flow measure, and goal orientations as a dispositional measure. With no inference of causality based on the method followed, it does seem that the athlete’s goal orientations can have either a positive influence or a detrimental effect on positive experience. The current study indicated that athletes’ task orientations may be the critical factor for attaining flow in competitive sport, since athletes in high task quadrants, irrespectively of ego orientation level, revealed the most positive experiential characteristics. Further to that, based on present findings it can be proposed that the balance or the ratio between athlete’s goal orientations is more important for the formulation of flow experience rather than the independent relationship of the dominant goal orientation type to positive emotions. It seems that task oriented athletes (high task-high ego and high task-low ego oriented ones) feel more skillful and they are estimating the upcoming competition as challenging. Possible explicative reasons behind this finding could include previously presented suggestions linking task orientation to lower anxiety levels, enhanced intrinsic motivation vignettes and adaptive psychological profiles (Duda, 1992, 2001). Conversely, ego oriented athletes (HE-LT oriented ones) lack important elements for getting into flow. The findings provide important information to coaches and sport psychology consultants, and may be of use in formulating psychological preparation programs that will foster the experience of flow and, facilitate athletes’ performance. Using various instruments, as well as, different research approaches similarly to the present study, will be helpful to capture, understand, and interpret the competitive experience from an athlete’s perspective.

## Conflict of Interest Statement

The authors declare that the research was conducted in the absence of any commercial or financial relationships that could be construed as a potential conflict of interest.
